# Development of Hybrid IgG-Aptamer Sandwich Immunoassay Platform for Aflatoxin B1 Detection and Its Evaluation Onto Various Field Samples

**DOI:** 10.3389/fphar.2018.00271

**Published:** 2018-03-27

**Authors:** Y. V. V. Aswani Kumar, R. M. Renuka, Jayakrishnan Achuth, M. Venkataramana, M. Ushakiranmayi, P. Sudhakar

**Affiliations:** ^1^Department of Biotechnology, Acharya Nagarjuna University, Guntur, India; ^2^DRDO-BU-CLS, Bharathiar University, Coimbatore, India; ^3^Department of Botany and Microbiology, Acharya Nagarjuna University, Guntur, India

**Keywords:** aflatoxin B1, aptamer, immuno-column SELEX, IgG antibody, ALISA

## Abstract

The present study was aimed to develop a novel antibody-aptamer based hybrid detection strategy for specific and sensitive detection of aflatoxin B1 (AFB1) from contaminated food grains. The study comprises generation of ssDNA aptamers and anti-AFB1 IgG against AFB1 toxin. The generated bio-probes (aptamers and antibodies) were further characterized for their specificity and sensitivity using indirect ELISA. The generated aptamers namely AFB1a and AFB1b showed prominent reactivity and selectivity against AFB1 toxin. These aptamers were further characterized for their secondary structures and dG values were determined as −4.6 and −2.75 Kcal/mol, respectively. The detection limit (LOD) of AFB1a and anti-AFB1 IgG was determined as 5 and 10 ng/mL, respectively. The characterized aptamers and antibodies against AFB1 were used to develop the sandwich immunoassay. Anti AFB1 IgG was used as a capturing antibody whereas anti-AFB1a aptamer was used as its revealing partner in the assay. The limit of detection (LOD) of the immunoassay was determined to be 5 ng/mL of AFB1 standard toxin and showed no cross-reactivity with closely related mycotoxins. To assess the reliability of the developed method, several field samples contaminated with aflatoxin B1 was included in the study and results were validated with commercial AFB1-ELISA Kit. Additionally, the spiking studies were also carried out to demonstrate the consistency and dependability of the developed hybrid sandwich immunoassay wherein the toxins recovered were found to be ranging between 73 and 98.80% with the LOD at 5 ng/mL. In conclusion, the developed method may find the better utility in routine food testing laboratories for assessment of AFB1.

## Introduction

Mycotoxins are low molecular weight (MW ~700 Da) toxic secondary metabolites produced by filamentous fungi. Significant groups of mycotoxins present in food are aflatoxins (AF) (*Aspergillus* sp.,), ochratoxin A (OTA) (*Aspergillus* sp.*, and Penicillium* sp.,), trichothecenes, zearalenone and fumonisins B1 and B2 (FM) (*Fusarium* sp.,) (Kalagatur et al., 2017). As per the report of Food and Agricultural Organization, it is estimated that 25% of world's food crops are contaminated with mycotoxins and many studies reported high levels of mycotoxins in food grains (Alkadri et al., 2014; Arroyo-Manzanares et al., 2014; Zhao et al., 2017). Among the reported mycotoxins, Aflatoxins are well known toxins produced by *A. flavus* and *A. paraciticus*. Till date, 20 different types of aflatoxins reported, out of which four major aflatoxins are commonly found in food commodities namely aflatoxins B1, B2, G1, and G2 (Guo et al., 2014). Aflatoxins are known hepatocarcinogens and also induce mutagenic, teratogenic, estrogenic, haemorrhagic, nephrotoxic, neurotoxic and immunosuppressive effects in humans and farm animals (Mudili et al., 2014; Aiko and Mehta, 2016; Dai et al., 2017). Aflatoxin B1 (AFB1) has been placed as major Class I human carcinogen by IARC (International Agency for Research on Cancer) and is approximately 3-fold toxic than the other mycotoxins. Aflatoxin contamination in crops occurs due to high temperatures (27–38°C) and relative humidity (85%) that favors *Aspergillus* growth in the field. The post-harvest contamination by aflatoxin occurs due to the prevalence of moisture content favoring mold growth and inappropriate agricultural practices (Torres et al., 2014; Gacem and El Hadj-Khelil, 2016; Kalagatur et al., 2018).

Due to the deleterious effects of Aflatoxins on humans and animals, many countries have proposed stringent regulations and allowed limits on cereals and food products intended for consumption. In accordance with global scenario, nearly 50 countries have already established legislation to monitor maximum AFB1 threshold limits in food products. International Agency for Research on Cancer (IARC) limits AFB1 in food and feeds up to 1–20 and 0–50 ppb, respectively (FSSAI, 2015). European Pharmacopeia permits AFB1 to 2 μg/kg and total aflatoxins to 4 μg/kg in herbal drug formulations, also 2 ppb AFB1 and 4 ppb total aflatoxins in cereal products. The guidelines set by Food Safety and Standards Authority of India permits aflatoxins to 15 and 30 ppb in cereal and spices, respectively. However, most of the study reports suggesting that there is a presence of huge quantities of these toxins in food grains which are utilizing for food and feed production. This could be the main reason for the lack of proper rapid and low-cost detection systems against these toxins in India. The commercially available systems are more costly and difficult to get in the resource-poor settings in developing and underdeveloped countries. Conventional analytical techniques including HPLC, GC-MS, LC-MS are though highly sensitive for mycotoxin quantification but the sophisticated instrumentation and tedious sample preparation restricts its onsite application (Suri et al., 2008; Turner et al., 2015). Immunoassay techniques such as Enzyme Linked Immuno Sorbent Assays (ELISA), fluorescence polarization immunoassay, immunochromatographic assay, and immuno sensors have paved the potential path for development of rapid mycotoxin detection systems (Suri et al., 2009; Venkataramana et al., 2015; Bintvihok et al., 2016; Jafari et al., 2017). In addition to being highly specific, they also show desirable sensitivity and specificity for the detection of low molecular toxic compounds (Sharma et al., 2010; Tey et al., 2010). However, antibody instability in different environmental conditions and high production cost restricts its applications (Shim et al., 2014a,b). Moreover, antibodies generation against low molecular weight toxin molecules is a rigid task since low molecular weight compounds non-immunogenic and specific antibodies production when administered to the immune system of a host animal is not significant (Gandhi et al., 2009). Hence, there is an immediate need to develop a low-cost and rapid detection method for onsite detection of aflatoxins from contaminated food grains.

In recent years, nucleic-acid (Aptamer) based immune assays have drawn attention toward diagnostic utility. Aptamers are oligonucleotides fragments, such as ribonucleic acid (RNA) and single-strand deoxyribonucleic acid (ssDNA) or peptide molecules with high specificity and affinity toward the target (Alkadri et al., 2014). The target-specific ssDNA was isolated from combinatorial libraries of synthetic nucleic acid by exponential enrichment via an iterative process of adsorption, recovery and amplification are known as Systematic Evolution of Ligands by Exponential enrichment (SELEX) (Venkataramana et al., 2015). The high binding affinities toward target molecules along with high selectivity pave the way for potential application toward bio-sensing platform development (Mudili et al., 2015; Nezlin, 2016).

The present study emphasis on the generation of specific ssDNA Aptamers against AFB1 by immuno-affinity column (IA column) based SELEX methodology and anti AFB1-rabbit IgG were generated. To make the generated Aptamers more specific, other Mycotoxins including series of other Aflatoxins (AFB2.AFG1, AFG2), Ochratoxin (OTA), Deoxynivalenol (DON), citrinin, and Fumonisin B1 (FB1) was included in the study for counter SELEX process. Selected target specific high reactive apatmers were sequenced and assessed for its secondary structures and dissociation constants (KD values) M-fold bioinformatics tool (Zuker, 2003). Generated aptamers and IgG were further used develop the sandwich assay for sensitive, selective and low-cost detection of AFB1 from contaminated food grains. The developed method was screened against several artificially contaminated as well naturally contaminated food samples and same results were co-evaluated with commercially available Aflatoxin ELISA Kit (MyBioSource, US; MBS702574).

## Materials and methods

### Chemicals, media, and reagents

All the salts were purchased from Hi-media Laboratories (India), except mentioned specifically. The aptamer library, primers and biotinylated probes were synthesized at 1 mM and 25 nM scale, respectively at IDT (US). The stock and working dilutions of the library and the primers were maintained in MilliQ water. The Taq DNA polymerase and PCR buffers were purchased from Sigma and pGEM-T vector and T4 DNA ligation enzymes were purchased from Promega (US).

### DNA library and primers

The ssDNA library (1,000 nmol) with 45 nucleotides comprising central random regions flanked by constant regions at 3′ and 5′ ends was purchased from IDT technologies (US). Forward and reverse primers (Table 1) were used to amplify the pool and biotinylated reverse primer was employed to label aptamers.

**Table 1:** Primers used in Aptamers generation and sequence selected aptamers (AFB1a and AFB1b).

**Name**	**Sequence (5′-3′)**	**Synthesis scale**
SelexAPLIB2	ATAGGAGTCACGACGACCAGAANNNNNNNNNNNNNNNNNNNNNNNNNNNNNNNNNNNNNNNNTATGTGCGTCTACCTCTTGACTAAT	1 μM
Apta F1 N	ATAGGAGTCACGACGACCAGAA	250 nM
Apta R1 N	ATTAGTCAAGAGGTAGACGCACATA	250 nM
Apt Bio Rev	5Biosg/ATTAGTCAAGAGGTAGACGCACATA	250 nM
**SELECTED APTAMERS SEQUENCES AFTER DIFFERENT ROUNDS OF SELEX**		
AFB1a	TATAGGAGTCACGACGACCAGAAAGTAATGCCCGGTAGTTATTCAAAGATGAGTAGGAAAAGATATGTGCGTCTACCTCTTGACTA	
AFB1b	ATAGGAGTCACGACGACCAGAAAGTAATGCCCGGTAGTTATTCAAAGATGAGTAGGAAAGATATGTGCGTCTACCTCTTGACTA	

### Generation and characterization of AFB1 aptamers

#### Generation of anti AFB1 aptamers

**Figure 1:**
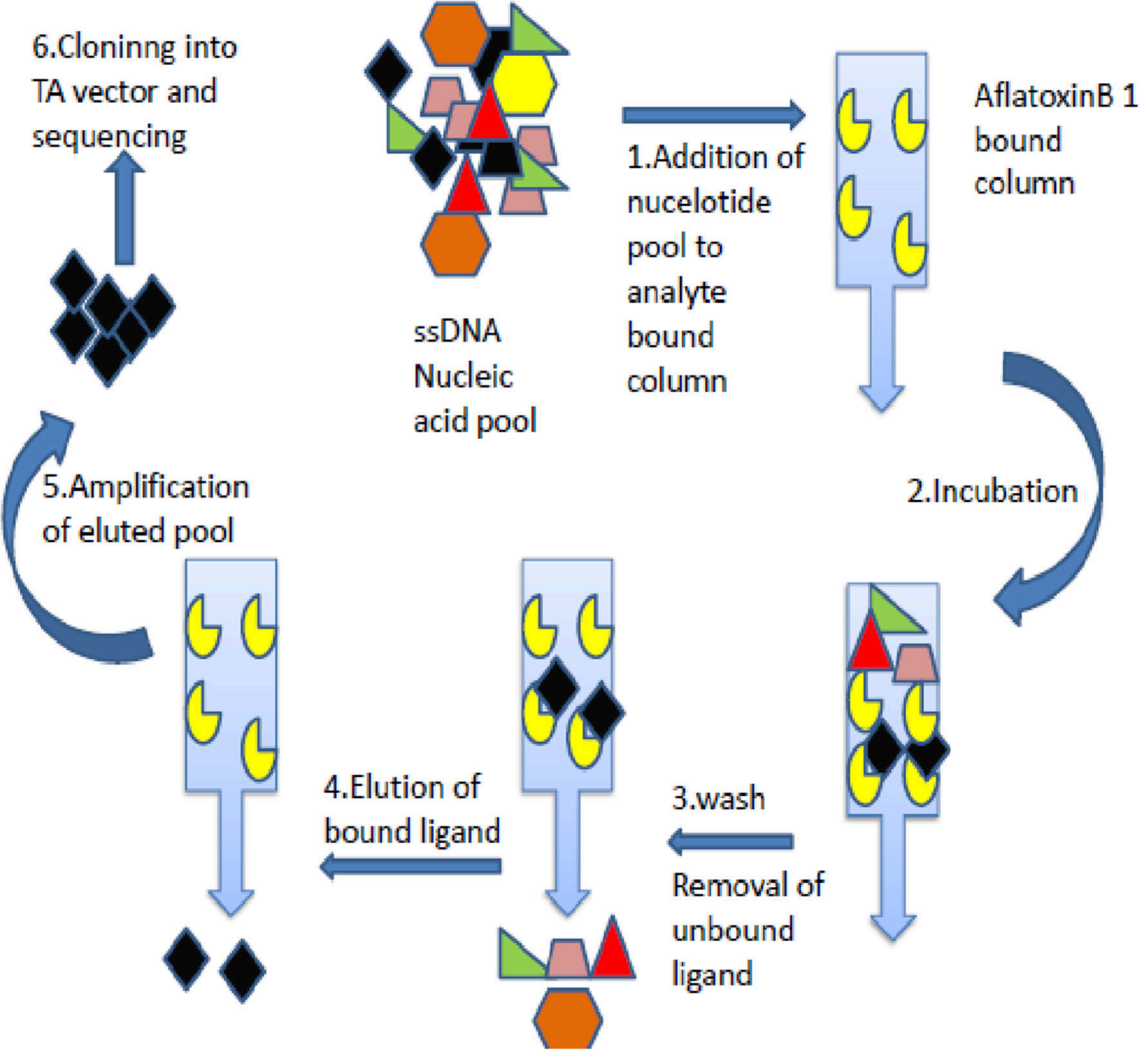
Schematic depiction of aptamers selection procedure. The figure describes Immuno- SELEX (Systematic evolution of ligand by exponential enrichment) technology for AflatoxinB1 specific aptamer generation.

The AFB1 specific aptamers were generated through SELEX technology employing anti AFB1 immunoaffinity column matrix (Figure 1). Briefly, Afla-clean Immuno Affinity column was equilibrated with binding buffer [(50 mM Tris-Cl (pH7.4), 5 mM KCl, 100 mM NaCl, 1 mM MgCl_2_] and incubated with the crude toxin (100 ng/μL) for 15 min. The flow-through was added onto the column and cycle was repeated 5–6 times to enhance binding efficiency. The toxin immobilized columns were incubated with single-stranded DNA oligos (100–200 pmol, pre-warmed @ 95°C) at room temperature for 1 h. The flow-through was collected and columns washed with binding buffer to remove unbound residues. The bound oligonucleotides were eluted with 100 μL of 1 × PCR buffer (pre-warmed @ 95°C). The eluted fraction was added with an equal volume of isopropanol and incubated at −20°C for 1 h. The solution was centrifuged and pellet thus obtained was air dried and resuspended in 25–30 μL of water (molecular biology grade-Himedia). The PCR reaction was set with obtained DNA (denaturation for 15 s at 95°C, the annealing temperature of 57°C for 30 s and extension at 72°C for 45 s with 30 cycles each.) and the amplified products thus obtained were subjected to further selection rounds. In total, 5 rounds of SELEX and 3 rounds of counter SELEX were performed and the ssDNA aptamers reactive against AFB1 were generated. The aptamer pools were cloned into pGEM-T vectors with TA cloning kit (Promega, US) and highly reactive clones were sequenced. In brief, the generated aptamer pool was amplified with Taq DNA polymerase with additional final extension duration. The amplified aptamers were ligated with pGEM-T vector and transformed into *E. coli* DH5α cells. Clones obtained were screened with M13 forward and reverse primers by colony PCR and positive clones with high specificity toward AFB1 were sequenced. The M fold software was used to predict secondary structure and aptamer with least dissociation constant was used for further studies.

#### Characterization of AFB1a aptamer

The AFB1a titer values were determined by indirect ELISA onto microtiter plates. Briefly, AFB1-Ovalbumin toxin (1 μg/well) in carbonate-bicarbonate buffer (pH 9.6) were coated onto the microtiter plates and incubated at 37°C for 1 h. The unbound sites were blocked with 3% Bovine serum albumin (BSA) in Phosphate Buffered Saline (PBS) (w/v) and biotinylated AFB1a aptamers following 2-fold dilution from 1:200 to 1:51,200 in PBS and incubated at 37°C for 1 h. The plates were washed with Phosphate Buffered Saline with Tween (PBST) thrice followed by PBS and incubated with streptavidin-HRP conjugate (1:2,000 dilutions) at 37°C for 1 h. The plates were washed and developed with OPD (Genie, Bangalore) followed by OD_450_. The specificity of the generated ssDNA aptamers was determined by DOT ELISA method. AFB1-OVA toxin (10 μg) was coated onto a nitrocellulose membrane, PBS (negative control), AFB2, AFB G1 &G2, Ochratoxin A, Citrinin, Deoxynivalenol, and Fumonisin B1 conjugated with Ovalbumin (OVA) protein. The membrane was blocked with 5% milk solution (w/v) in PBS for 2 h at 37°C. Further, the membrane was washed with 1 × PBST and 1 × PBS thrice each and incubated with biotinylated aptamer (1:1,000 dilution, denatured and flash frozen) at 37°C for 1 h. The membrane was washed as above mentioned and was incubated with streptavidin-HRP conjugate (1:2,000 dilutions) for 1 h. The membrane was subsequently washed and developed with TMB-H_2_O_2_ (Genie, Bangalore). The AFB1-OVA concentration from 10 μg/μl to −0.001 μg/μl were coated onto microtiter plates and blocked with 3% BSA in PBS. The indirect ELISA protocol was followed as described above with AFB1a aptamers. The values were read at 450 nm after development. PBS was used as negative control for all the assays described above.

### Generation AFB1 specific antibodies

#### Preparation of AFB1 protein conjugates and generation of anti AFB1 antibodies

The amino group's activation for protein carriers (Ovalbumin and BSA) were proceeded via ethylenediamine (EDA) with 1-Ethyl-3-[3-dimethylaminopropyl]-carbodiimide hydrochloride (EDC) procedure. The residual EDA and EDC were removed through dialysis against distilled water. AFB1-protein conjugate was washed with 0.1 M morpholineethanesulfonic buffer (MES) (pH 4.8) five times followed by purification through 10 kDa MWCO centrifugal filters and the aliquots stored at −20°C. The AFB1-BSA conjugate (100 μg/ mL) with Freund's complete adjuvant (Difco, US) was immunized intramuscularly to white New Zealand rabbits. Pre-immunized serum was collected and stored until till further assay as a negative control. After 14 days of primary dosage, booster dosages (AFB1-150 μg/ mL) in Freund's incomplete adjuvant were immunized (Wijaya et al., 2010) (Difco, US). Serum was collected and titer value estimated by indirect ELISA against Aflatoxin-Ovalbumin conjugate (1 μg/well) as antigen as mentioned above. The rabbits were subsequently immunized with AFB1-BSA emulsified in incomplete adjuvant at 15 days interval till an endpoint titer value of 1:64,000 (ANUCPS/IAEC/AH/Protocol/2/2014: Dt 15/07/2014).

#### Purification of anti-AFB1 IgG antibodies

The hyper-immune rabbits were bled through ear veins and separated serum was diluted in acetate buffer (sodium acetate 0.147 g, acetic acid 295 μl, D/W 100 ml, pH 4.5) at ratio of 1:5. To the solution, 250 μl of caprylic acid was added under constant stirring and centrifuged at 10,000 g for 30 min at room temperature. The supernatant was filtered and added with saturated ammonium sulfate solution followed by incubation for 30 min at room temperature. The solution was centrifuged at 10,000 g for 15 min at 4°C and pellets were dissolved in PBS (1:2). The precipitate was dialyzed against PBS at 4°C overnight.

#### Specificity and sensitivity evaluation of anti-AFB1 IgG antibody

The anti-AFB1 IgG titre value was determined by indirect ELISA. In brief, the AFB1-OVA (1 μg/well) coated plates were blocked with 3% BSA (W/V in PBS). The anti AFB1 with 2-fold dilutions from 1:100 to 1:28,000 in PBS were added to the representative wells and incubated at 37°C for 1 h. The plates were washed successively with PBST and PBS followed by incubation with anti-rabbit HRP (1:2,000) at 37°C for 1 h. The plates were washed as above and developed with OPD followed by OD at 450 nm. The specificity of anti-AFB1 IgG was determined by indirect-ELISA method against AFB2, AFB G1 &G2, OTA, Citrinin, DON, and FB1 conjugated with OVA protein. The sensitivity of anti-AFB1 IgG was also determined against various AFB1-OVA toxin concentrations from 10 to 0.1 μg/μl by indirect ELISA method as given above. PBS was used as negative control for all the assays described above.

### Development and characterization of aptamer-IgG based sandwich ALISA

The aptamer- antibodies raised against AFB1 was applied to develop sandwich hybrid immunoassay. Anti-AFB1 IgG was employed as capturing antibody and ssDNA AFB1 aptamer as its revealing entity.Anti-AFB1 IgG (300 ng/mL) in carbonate-bicarbonate buffer (pH 9.6) were coated onto microtiter plates and incubated at 37°C for 1 h. The plates were blocked with 3% BSA (w/v in PBS) at 37°C for 1 h and washed with PBST and PBS thrice, successively. AFB1-OVA conjugates with varying concentration (10–0.1 μg/μl) were added and incubated at 37°C for 1 h. The plates were washed thrice with PBST and PBS followed by incubation with biotinylated AFB1a aptamer 37°C for 1 h. The secondary conjugate (streptavidin –HRP; 1:2,000 dilutions in PBS) were added post washing and incubated at 37°C for 1 h. The plates were washed, followed by development with OPD substrate and samples were read at 450 nm. The assay was further characterized for specificity by dot-ELISA against toxins AFB2, AFB G1 & G2, OTA, Citrinin, DON, and FB1 conjugated with ovalbumin protein. PBS was used as negative control for all the assays described above.

### Aflatoxin B1 extraction from fungal cultures and contaminated food samples

Fungal cultures comprising *Aspergillus flavus, Aspergillus parasiticus, Aspergillus niger, Fusarium sporotrichoides, Fusarium graminearum*, and *Penicillium chrysogenum* were cultured on potato dextrose broth (Hi-media, India) The mycelium was filtered and resuspended in Czapek yeast extract and incubated at 30°C for 10 days at 150 rpm to facilitate the toxin production. Mycelium thus obtained was filtered and toxins were extracted with 25 ml of ethyl acetate. Ethyl acetate was removed by evaporation and mycotoxins were resuspended in methanol and stored at 4°C until further use (Solfrizzo et al., 1998; Priyanka et al., 2014).

To access the usability and reliability of the developed immunoassay, cereal samples, maize and corn were collected from various storage houses and farming fields from different regions of southern India. AFB1 was extracted from cereal samples according to the methodology described by Solfrizzo et al. (1998). Briefly, samples were finely grounded and 50 g was preceded by toxin extraction. The samples were mixed with chloroform, 0.1 M phosphoric acid and incubated for 30 min on a rotary shaker. The mixture was filtered through Whatman no. 1 paper and subjected to evaporation on a rotary evaporator. The collected residue was reconstituted with 1 ml of acetic acid-water- acetonitrile (1:58:41). The extract was resuspended in hexane through continuous vortexing and centrifuged at 10,000 rpm for 10 min. The lower phase was filtered through 0.45 μm syringe filter and sample thus obtained were preceded for developed sandwich immunoassay. The samples were assessed with developed immunoassay sandwich ELISA and commercially available Aflatoxin ELISA Kit (MyBioSource, US).

To evaluate the toxin recovery rate from different food matrices, standard AFB1 (20–1 ng) toxin was spiked on to maize, paddy, corn and groundnut samples. AFB1 extraction from spiked samples was carried out following Stroka et al. (1999) and retained toxin extracts was air dried and resuspended in 1X PBS (500 μl). The recovered toxin was analyzed with the developed hybrid sandwich immunoassay. The concentration of the spiked AFB1 toxin recovered from different food matrices was assessed by plotting measured optical density (nm) against various toxin concentrations employing linear regression (*R*^2^ = 0.9958). The concentration of AFB1 recovered thus obtained was employed for determining the recovery percentage from the following formula.

$$\begin{array}{l} \%\text{ recovery}=\frac{Concentration\;of\;AFB1\;spiked\;-Concentration\;of\;AFB1\;recovered}{Concetration\;of\;AFB1\;spiked}\;\times \;100 \end{array}$$

All experiments were performed in triplicates with defined conditions to ensure the reliability of data. Statistical differences between treatments were analyzed by univariate (ANOVA) and *t*-test assuming *p*-value < 0.05 and plotted using Graph Pad Prism 6.

## Results

### Generation and characterization of AFB1 aptamers

#### Generation of AFB1 aptamers

**Figure 2:**
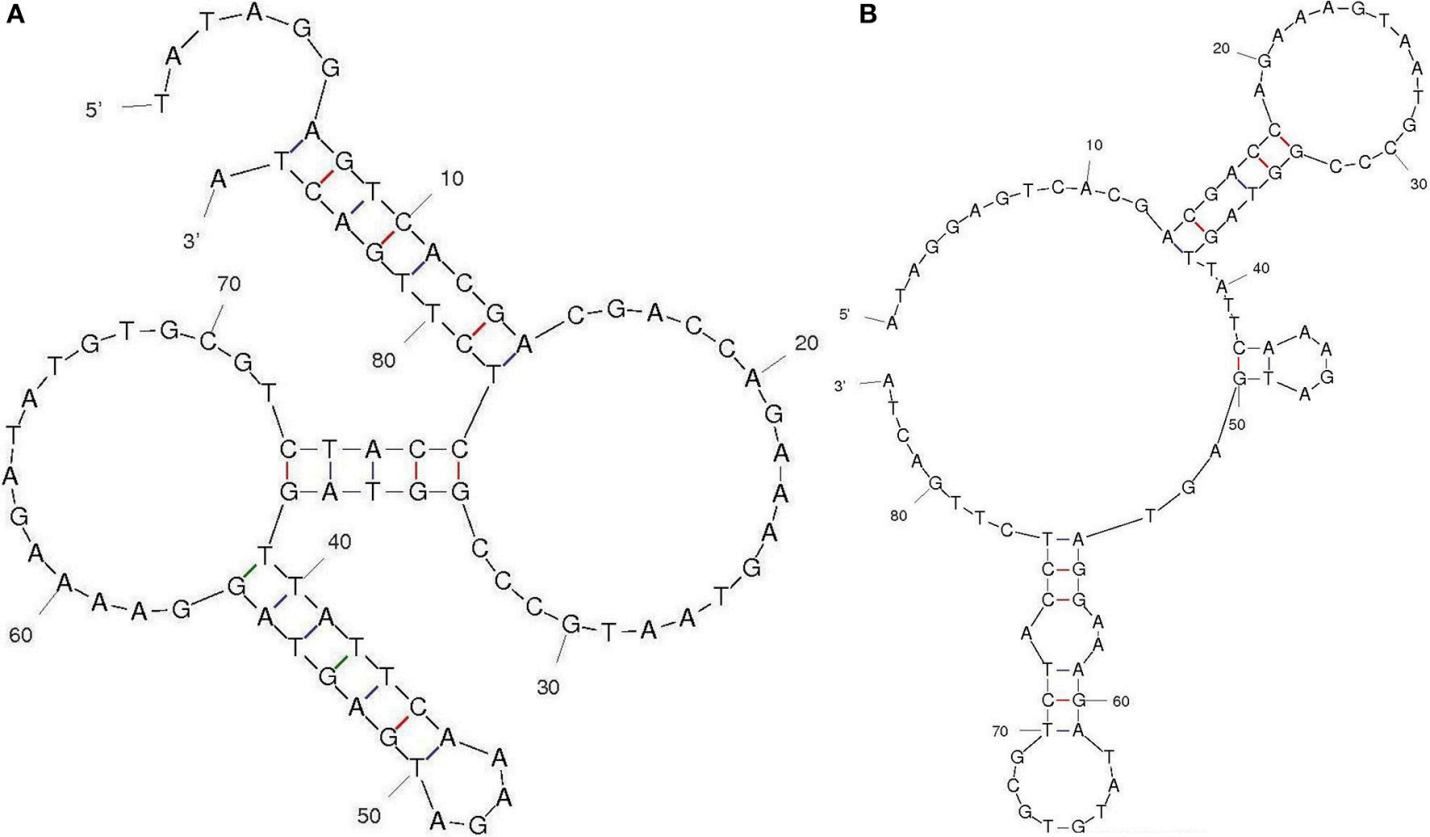
Aptamer secondary structure. Selected aptamer secondary structures and dissociation constant predicted through M-fold software **(A)** AFB1a and **(B)** AFB1b.

Aptamers specific to Aflatoxin B1 was generated by immunoaffinity column based SELEX with five and three rounds of SELEX and counter SELEX respectively. The un-reactive pools/non-specific pools accumulation was eliminated through stringent wash steps as well as counter SELEX. Hence the final pool thus obtained consisted highly specific aptamers which were subjected to asymmetric PCR with increased extension time (15 min), to acquire poly A tagging at both ends. Amplified aptamer pools were cloned into a pGMET cloning vector, transformed positive colonies for target insert were recovered and plasmids were purified and sequenced for target Aptamer (AFB1a and b) sequences (Table 1). M-Fold server was used to predict the secondary structures and free energy calculations. Predicted secondary structures were shown in Figure 2, and the free energy of AFB1a and AFB1b were determined as −4.6 and −2.75 Kcal/mol respectively. Due to its higher binding efficiency and selectivity AFB1a aptamer was used for diagnostic assay development.

#### Characterization of selected AFB1a aptamer

**Figure 3:**
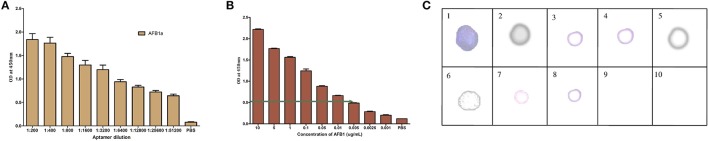
Characterization of selected AFB1a aptamer. **(A)** The AFB1a aptamer titre values determined through indirect ELISA. **(B)** Limit of detection analysis by indirect ELISA of AFB1a aptamer against various AFB1 concentration from 10–0.001 μg/mL. **(C)** Specificity analysis of AFB1a aptamer determined by dot-ALISA, 1-AFB1, 2-AFB2, 3-AFG1, 4-AFG2, 5-Ochratoxin A, 6- Citrinin, 7-DON, 8-FB1, 9, and 10-BLANK.

The titer values of AFB1a aptamer was determined by indirect ELISA against AFB1-OVA conjugate and was found to be 1:5,12,000 dilutions (Figure 3A). The obtained dilution corresponds to 150 ng/mL of the AFB1a aptamer concentration and the same was employed for the further assays. The specificity assessment of the AFB1a Aptamer by dot-ELISA technique revealed that AFB1a aptamers detects AFB1 toxin specifically and showed no cross-reactivity against AFB G1 &G2, OTA, Citrinin, DON, and FB1 (Figure 3C). AFB2 showed cross-reactivity against AFB1a but significantly lesser than the AFB1. AFB1a sensitivity determined against varying AFB1 standard toxin concentration (10–0.001 μg/mL) by indirect ELISA technique showed the limit of detection (LOD) up to 0.005 μg (Figure 3B).

### Generation and characterization anti-AFB1 antibodies

#### Generation and purification of anti-AFB1 antibodies

The molar ratio 1:100 was used in the conjugation of AFB1 with the carrier proteins (Ovalbumin and Bovine Serum Albumin) (Gandhi et al., 2008; Venkataramana et al., 2015). AFB1-protein conjugation was achieved by condensation of cationized proteins with formaldehyde followed by reaction with α-hydrogen adjacent carbonyl in AFB1. The decrease in fee lysine residues corresponds directly to unavailable amino group hence indirectly corresponding covalent bond formation between the AFB1 and carrier proteins (Aiko and Mehta, 2016). AFB1-BSA and AFB1-OVA conjugates were employed respectively for immunization and coating antigen. The anti-AFB1 antibodies raised in rabbit against AFB1-BSA immunization was obtained from sera through ear vein bleeding and purified by caprylic acid precipitation method.

#### Characterization of anti-AFB1 antibodies

**Figure 4:**
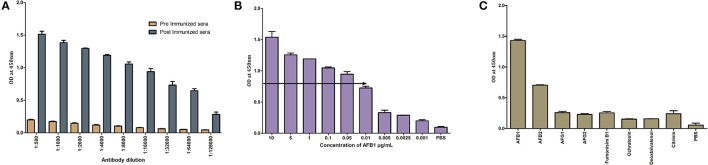
Characterization of generated anti- AFB1 antibody. **(A)** Indirect ELISA titre value determination of anti-AFB1 antibody. **(B)** Anti-AFB1 antibody limit of detection analysis against different AFB1 concentration (10–0.001 μg/mL) by indirect ELISA. **(C)** Cross specificity analysis of anti-AFB1 antibody against 1-AFB1, 2-AFB2, 3-AFG1, 4-AFG2, 5-Ochratoxin A, 6-Citrinin, 7-DON, 8-FB1, 9 PBS.

Titer values of anti-AFB1 determined by indirect ELISA against AFB1-OVA (1 μg/mL) antigen and was found to be 1: 64,000 (Figure 4A). The titer value corresponds to an antibody concentration of 300 ng/mL and the same was implemented in further assays. The sensitivity analysis determined by indirect ELISA against AFB1-OVA toxin (10–0.001 μg/ mL) showed the LOD up to 0.01 μg for anti-AFB1 antibody (Figure 4B). Anti-AFB1 antibody showed no cross-reactivity against AFB G1 &G2, OTA, Citrinin, DON, and FB1 (Figure 4C). And AFB2 toxin was found to cross-react with anti-AFB1 antibodies.

### Development and characterization of aptamer-antibody hybrid sandwich immunoassay technique

**Figure 5:**
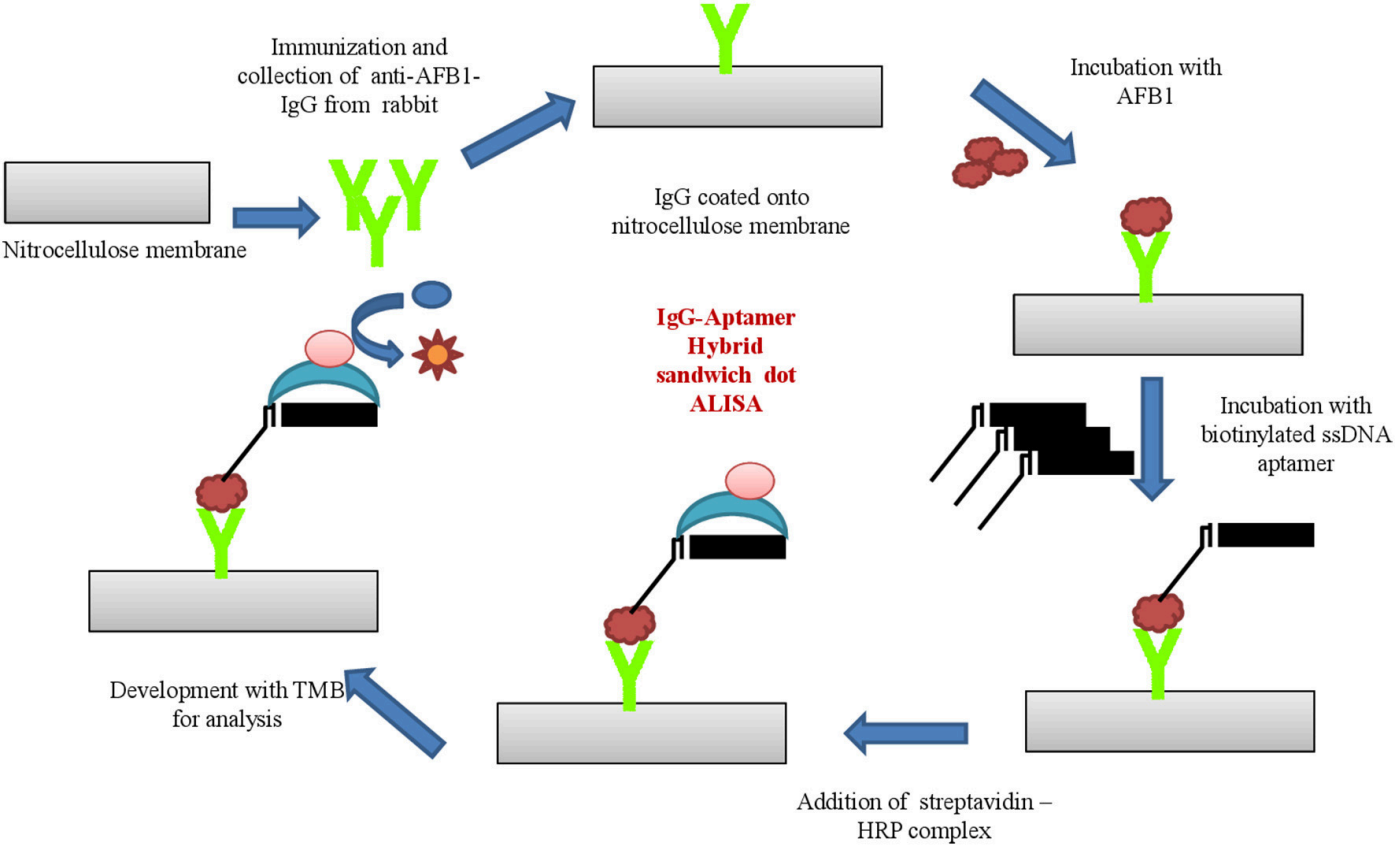
Schematic illustration of developed Aptamer-antibody sandwich immunoassay.

**Figure 6:**
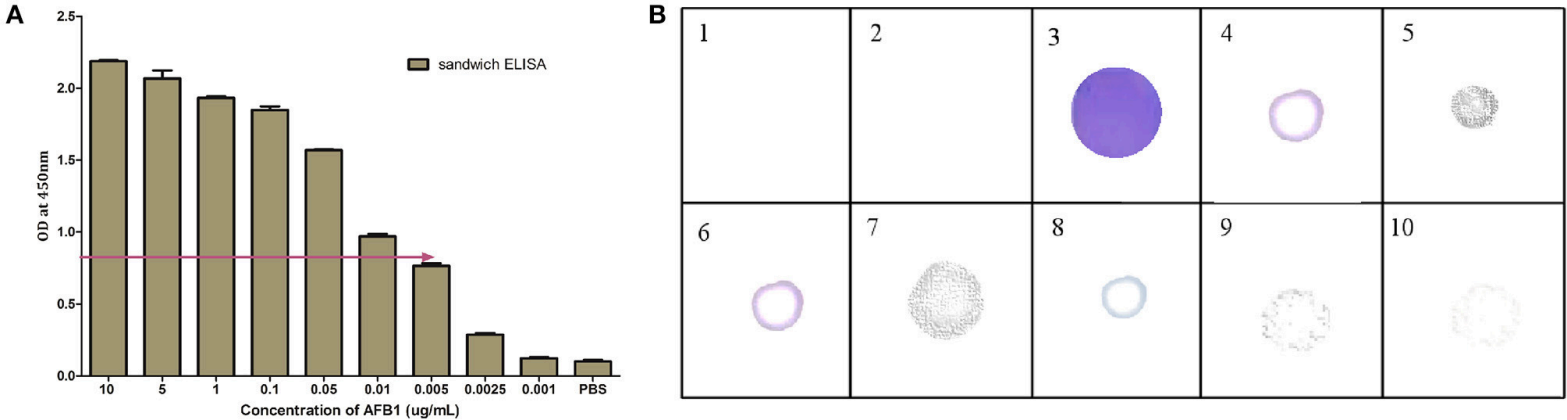
Characterization of AFB1a Aptamer-anti-AFB1 antibody based sandwich immunoassay: **(A)** Limit of detection for developed sandwich ALISA platform determined by indirect ELISA against different AFB1 concentration (10–0.001 μg/mL). **(B)** Cross reactivity analysis of developed sandwich immunoassay against 1 and 2-Blank, 3-AFB1, 4-AFB2, 5-AFG1, 6-AFG2, 7-Ochratoxin A, 8- Citrinin, 9-DON, 10-FB1.

Based on the above results, the generated bio probes (AFB1a aptamer and anti-AFB1 IgG) were employed to develop sandwich hybrid immunoassay (Figure 5). The anti AFB1 antibody was selected as the capturing probe and AFB1a aptamer as its revealing probe. The generated AFB1a aptamer (150 ng/ mL) and anti-AFB1 antibody (300 ng/ mL) were found to be sensitive up to 0.005 μg (5 ng) against AFB1-OVA target (Figure 6A). The immunoassay showed no cross-reactivity against, AFB G1 &G2, OTA, Citrinin, DON, and FB1 and showed cross-reactivity with AFB2 (Figure 6B).

### Evaluation of developed sandwich immunoassay

**Figure 7:**
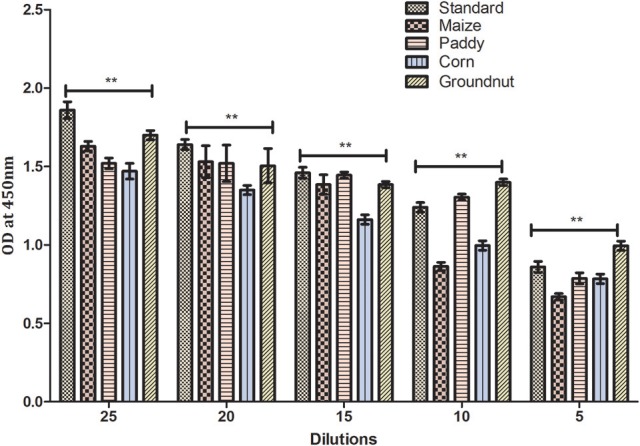
Matrix interference of developed hybrid sandwich immunoassay. The matrix inference assay of various field samples spiked with AFB1 toxin (25–5 ng). Asterisks indicate a comparison between standard, maize, paddy, corn and groundnut samples, **p < 0.01 of independent experiments performed and statistically evaluated using ANOVA.

Results of the developed method were equivocally matched with result obtained using commercially available Aflatoxin ELISA Kit (MyBioSource, US) (Table 2). To demonstrate the prospective application of the developed hybrid sandwich immunoassay, spiking study was conducted using maize, paddy, corn and groundnut samples. A standard curve was plotted with measured optical density (nm) against various toxin concentrations. Linear correlation (*R*^2^ = 0.9958) was obtained and recovery percentage were drawn for spiked samples namely maize (96.5–73%), paddy (95.2–76.8%), corn (93.6–85%), and groundnut (98.8–93.4%) (Table 3). From the matrix interference assay performed using the developed sandwich immunoassay platform showed a *p*-value of < 0.05 thus statistically significant demonstrating the lack of interferences by matrices (Figure 7). Thus the developed sandwich immunoassay platform can be successfully employed in the detection and purification of Aflatoxin B1 from food and agro commodities.

**Table 2:** Evaluation of sandwich immunoassay against standard cultures and field samples.

**S. No**	**Strain name**	**Standard ELISA Kit**	**Sandwich ALISA**
1	*Aspergillus flavus* NCIM 152	+	+
2	*Aspergillus flavus* ATCC 46283	+	+
3	*Aspergillus flavus* NCIM 650	+	+
4	*Aspergillus flavus* NCIM 645	+	+
5	*Aspergillus flavus* MTCC 2798	+	+
6	*Aspergillus Parasiticus* MTCC 2797	+	+
7	*Aspergillus Parasiticus* MTCC 898	+	+
8	*Aspergillus niger* MTCC 9687	−	−
9	*Penicillium chrysogenum* MTCC6479	−	−
10	*Fusarium sporotrichoides* MTCC 2081	−	−
11	*Fusarium graminearum* MTCC 2089	−	−
12	Maize samples 01	+	+
13	Maize samples 02	+	+
14	Maize samples 03	+	+
15	Maize samples 04	+	+
16	Maize samples 05	−	−
17	Maize samples 06	−	−
18	Maize samples 07	+	+
19	Maize samples 08	−	−
20	Paddy samples 01	+	+
21	Paddy samples 02	+	+
22	Paddy samples 03	−	−
23	Paddy samples 04	+	+
24	Paddy samples 05	−	−
25	Paddy samples 06	−	−
26	Paddy samples 07	+	+
27	Paddy samples 08	−	−
28	Ground nut 01	+	+
29	Ground nut 02	+	+
30	Ground nut 03	+	+
31	Ground nut 04	−	−
32	Ground nut 05	+	+
33	Ground nut 06	−	−
34	Ground nut 07	−	−
35	Ground nut 08	−	−
36	Corn samples 01	+	+
37	Corn samples 02	−	−
38	Corn samples 03	+	+
39	Corn samples 04	+	+
40	Corn samples 05	+	+
41	Corn samples 06	+	+
42	Corn samples 07	−	−
43	Corn samples 08	−	−

**Table 3:** Recovery result of aflatoxin B1 spiked food samples detected by developed hybrid sandwich assay.

**S. No**	**Spiked concentration (ng/ mL)**	**Detected concentration (ng/mL)**				**Recovery ratio (%)**			
		**Maize**	**Paddy**	**Corn**	**Groundnut**	**Maize**	**Paddy**	**Corn**	**Groundnut**
1	25	24.1	23.8	23.4	24.7	96.4	95.2	93.6	98.8
2	20	19.3	19.18	18.76	19.4	96.5	95.9	93.8	97
3	15	14.56	14.7	12.8	14.52	97	98	85.3	96.8
4	10	7.86	8.93	8.01	9.32	78.6	89.3	80	93.2
5	5	3.65	4.24	4.25	4.67	73	76.8	85	93.4
6	0	0	0	0	0	0	0	0	0

## Discussion

Mycotoxins such as aflatoxin, fumonisins and ochratoxins, since its discovery in early 1960's possess serious health threat to both humans and animals. Also, being ubiquitous in nature tends to pose higher contamination risk associated with food and agro-related commodities (Mukherjee et al., 2017). The high incidence rates of these mycotoxins were reported in developing countries like India, restricting not only to groundnut and maize samples but also in medicinal herbs and processed foods. The economic implications associated with mycotoxin contamination include detrimental crop yield and poor quality grains. Hence, there is an increased concern regarding food product safety and quality, whereupon stringent regulations are in need to monitor as well as fix threshold levels. The higher mycotoxin incidence amongst various states of India that includes Gujarat, Punjab, Bihar, Andhra Pradesh, Tamil Nadu, and Karnataka was reported (Priyanka et al., 2015). The conventional segregation techniques such as physical removal of contaminated/moldy grains, density based segregation reduce mycotoxin incidences in grains considerably.

Hence, the prevailing scenario demands sensitive aflatoxin B1 detection system to monitor and manage its levels in food grains. The most profoundly employed mycotoxins detection strategy involves analytical techniques such as TLC and HPLC (Kumar et al., 2016). Being sensitive in operation, these techniques require skilled manpower and cumbersome cleanup procedures. Associated drawbacks could be overcome through an immunodiagnostic technique that utilizes bio-probes for detection. The immunochemical methods that apply affinity-based detection strategies employ antibody-based platforms for AFB1 detection. Antibody-based format has several drawbacks that include tedious antibody generation process followed by skilled labor required for its mass production (Chen et al., 2014; Gandhi et al., 2018a,b). Nucleic acid-based approaches pave way for novel strategy that embodies a stable and economical alternative to the antibodies (Wandtke et al., 2015).

Aptamers are nucleic acid-based probes with high target affinity. They are ssDNA or RNA based oligonucleotides generated by the systemic evolution of ligands by exponential enrichment (SELEX) technology (Tuerk and Gold, 1990). Their unique secondary structure promotes efficient target binding with a high degree of specificity similar to monoclonal antibodies. Aptamer generation against toxic molecules with smaller size is comparatively easier than antibody production therein holding potential application as a possible alternative for the latter *in vivo* applications. Additionally, availability of sequence information helps in custom made aptamers synthesis against selected targets to replace requisite animal immunization required for antibody generations (Ma et al., 2014; Malhotra et al., 2014; Sharma et al., 2016). The aptamer-based diagnostics could be applied for rapid detection of pathogens, microbial toxins and other hazardous environmental toxicants in order to ensure human health and safety (Zhijiang et al., 2014).

Aflatoxin immobilization/conjugation onto different matrix involve complex chemical reactions that could alter target protein native conformation (Ma et al., 2015). In the present study, we report a modified SELEX approach exploiting columns in selection and enrichment of AFB1 specific aptamers through multiple rounds. The column anchors AFB1 specific antibodies that facilitate native AFB1 toxin immobilization thereupon reducing any conformational/structural loss (Setlem et al., 2016). Nearly, five rounds of direct SELEX were performed followed by subsequent cycles of counter SELEX. The counter SELEX reduces non-specific residues and therewith facilitates AFB1 target-specific aptamers enrichment. The final AFB1 specific pools were cloned into the pGEMT vector via TA cloning and high reactive clones were sequenced. Furthermore, the secondary structure and dissociation constants analysis with the obtained sequence revealed lower dG values of −4.6 and −2.75 kcal/mol for AFB1a and AFB1b, respectively. Amongst the two, AFB1a with lower dG values was selected for further studies. AFB1a aptamers showed little to no reactivity with other mycotoxins except AFB2. AFB2 showed less significant cross-reactivity toward AFB1a compared to AFB1 M. Moreover the sensitivity for the AFB1a was found to be 5 ng/mL. This provides sufficient data supporting AFB1a application in development aflatoxin detection strategy.

The AFB1 toxin being non-immunogenic (owing to its low molecular weight) was conjugated to carrier proteins such as OVA/BSA to elicit an appropriate immune response. Moreover, the absence of reactive groups in AFB1 for interaction was overcome by carboxyl group activation via EDC/NHS chemistry. This strategy assisted in covalent bonding of AFB1 with OVA and BSA through amide bonds (Venkataramana et al., 2015). The polyclonal antibodies were raised against AFB1-BSA by rabbit immunization and its reactivity toward AFB1 was assessed against AFB1-OVA conjugate to prevent BSA-specific antibodies interference. The titer values for the generated polyclonal anti-AFB1 IgG were found to be 1:64,000. The polyclonal anti-AFB1 IgG were determined to be specific toward AFB1 with negligible cross-reactivity amongst other closely related mycotoxins except for AFB2. The AFB2 toxin produced mild cross-reactivity toward anti-AFB1 IgG with respect to AFB1. The sensitivity of anti-AFB1 IgG was found to be 10 ng/ mL.

The generated aptamers/antibodies were employed in the development of a hybrid sandwich immunoassay for AFB1 toxin detection. The anti-AFB1 IgG were employed as capturing moiety over aptamers due to the structural rigidity as well as less cross-reactivity toward the AFB2 toxin. LOD of the developed platform was determined to be 5 ng/mL (0.005 μg/mL) of AFB1 standard toxin and no cross-reactivity associated with related mycotoxins such as AFG1 &G2, OTA, Citrinin, DON, and FB1 was observed. The immunoassay showed mild cross-reactivity toward AFB2 but was insignificant on par with AFB1. Moreover, individual recovery percentages for spiked samples were found to be maize (96.5–73%), paddy (95.2–76.8%), corn (93.6–85%), and groundnut samples (98.8–93.4%).

## Author contributions

YA and MV Designed the experiments. YA, JA, and RR performed experiments. MV, MU, and PS prepared the manuscript. PS and MV reviewed the manuscript.

### Conflict of interest statement

The authors declare that the research was conducted in the absence of any commercial or financial relationships that could be construed as a potential conflict of interest.
